# A meta-analysis of the relationship between body mass index and risk of rheumatoid arthritis

**DOI:** 10.17179/excli2018-1763

**Published:** 2018-11-06

**Authors:** Ying Zhou, Mingfang Sun

**Affiliations:** 1 Division of Rheumatology, Research Institute of Surgery, Daping Hospital, the Army Medical University, Chongqing 400042, China

**Keywords:** rheumatoid arthritis (RA), body mass index (BMI), meta-analysis, risk factor

## Abstract

The present meta-analysis aimed to evaluate the relationship between body mass index (BMI) and rheumatoid arthritis (RA). A systematic search of the Cochrane, Pubmed, and Embase databases was conducted to identify relevant studies published before September 2017 using terms related to BMI and RA. Fixed or random-effect models were used to estimate the pooled relative risk (RR) with 95 % confidence interval (CI). Subgroup analyses by sex were performed to investigate the association between BMI and RA in male and female subgroups. A total of 14 eligible studies containing 353,948 patients were included in the analysis. The pooled results suggested that the odds ratios (ORs) of RA were 1.08 (95 % CI: 1.00~1.15) for overweight, and 1.32 (95 % CI: 1.11~1.54) for obesity, respectively, suggesting that a higher BMI increases the risk of RA compared to normal weight. Further subgroup analyses showed a positive association between BMI and RA risk but only in females, with a RR of 1.11 (95 % CI: 1.00~1.22) for overweight and 1.40 (95 % CI: 1.24~1.57) for obesity. In conclusion, an increased BMI may lead to a higher risk for RA development. Furthermore, the positive association between BMI and RA risk may be stronger in female populations than in males. However, additional analyses are needed.

## Introduction

Rheumatoid arthritis (RA), a common chronic autoimmune disease, is characterized by chronic, destructive, debilitating arthritis (Gabriel et al. 1999[12]). The incidence rate of adult RA worldwide is about 1 %, but the proportion of infected women is far higher than that of men (Alamanos and Drosos, 2005[1]; Begovich et al., 2004[4]). Thus far, the exact etiology and pathogenic mechanisms underlying RA remain unknown, but interactions between genetic and environmental factors are generally accepted to be implicated in the susceptibility and severity of this complicated disorder. RA has been proven to have a familial component, which exerts about 15 % concordance and 53-65 % heritability in twin studies (MacGregor et al., 2000[21]; Silman et al., 1993[27]). Despite a high genetic susceptibility, the disease must also be influenced by environmental factors. The best support for this is the low concordance for RA in monozygotic twins (WHO, 2000[36]). Candidate risk factors that participate in the initiation of this disease include age, gender, obesity, cigarette smoking, hormone levels, psychological factors, alcohol consumption, and dietary habits, among others (Jin et al., 2014[17]; Parks et al., 2013[23]; Voigt et al., 1994[34]).

As the quality of life of the general population improves, obesity has become a common serious health problem affecting many people. The percent of overweight (body mass index (BMI) > 25 kg/m^2^) or obese (BMI > 30 kg/m^2^) individuals in the USA is estimated to be two thirds of the adult population and one third of children (Barry et al., 2012[2]). In general, being overweight, as determined by body mass index (BMI), is characterized by abnormal accumulation of adipose tissue that can accelerate the physiological and pathological processes associated with various inflammatory/autoimmune conditions due to immunomodulating and proinflammatory properties (Fantuzzi, 2005[10]; Versini et al., 2014[33]). Therefore, excess body weight may increase the risk of inflammatory diseases that have high morbidity and mortality, such as RA (Bastard et al., 2000[3]). According to relevant reports, more than 50 % of RA patients are accompanied by obesity (Uhlig et al., 1999[32]). In addition, numerous studies have suggested that obesity is a potential promoter in the development and progression of RA (Crowson et al., 2013[6]; Harpsøe et al., 2014[14]; Lahiri et al., 2014[18]; Ljung and Rantapää-Dahlqvist, 2016[19]; Symmons et al., 1997[30]; Uhlig et al., 1999[32]). This problem of obesity combined with the resulting metabolic syndrome in patients with RА has attracted extensive research attention (Symmons et al., 1997[30]).

Although many studies have focused on determining the association of body mass index (BMI) and RA risk, conflicting results have been obtained and clarification is needed. Several previous studies have proposed a positive correlation between high BMI and increased risk of RA development, suggesting that obesity is a key modifiable risk factor for RA (García-Poma et al., 2007[13]; Uhlig et al., 1999[32]). Conversely, other studies yielded contrary results (Cerhan et al., 2002[5]; Rodríguez et al., 2009[26]; Wesley et al., 2013[35]). Furthermore, some studies conclude that increased BMIs contribute to a poor RA outcomes (Hashimoto et al., 2009[15]), whereas others have reported that a high BMI may be associated with less radiographic joint damage (García-Poma et al., 2007[13]). To further determine the impact of BMI on the development of RA in a quantitative manner, as well as to explore BMI and the risk of RA in male and female subgroups, a meta-analysis was performed based on the available relevant literature.

## Methods

### Search strategy

To identify all eligible studies regarding the association between BMI and risk of RA, two investigators independently performed an electronic search in the Cochrane, Pubmed, and Embase databases up to September 2017. The MeSH terms were as follows: “Rheumatoid Arthritis” or “Rheumatoid Arthritis” combined with “Obesity” or “Obesity” or “Body Mass Index” or “Body Mass Index” or “Overweight”. All articles were limited to those published in the English language, but without other restrictions on ethnicity, geographic regions, or human subjects. The relevant articles from the initial search were further analyzed to identify the final qualified studies. If a disagreement occurred, it was resolved through the involvement of a third investigator.

### Inclusion and exclusion criteria

A study was included if it met the following criteria: (1) case-control or cohort design; (2) the exposures of interest were BMI, obesity, or overweight; (3) the prevalence of RA was defined by physicians or the record linkage system, which was deemed as the outcome of interest; (4) the data included relative risk (RR) or odds ratios (ORs) with 95 % confidence intervals (CIs); (5) published in English language. If more than one article was published on the same or overlapping populations, the most recent one with the largest sample size or most detailed information was selected. A study was excluded in case of: (1) repeating publications, or studies with similar content; (2) review, case report, theoretical research, mechanism studies, conference report, systematic review, meta-analysis, unpublished, expert comment, theoretical analysis, or non-human studies; (3) the BMI was not grouped by normal weight, overweight, and obesity classifications from the WHO; (4) did not have sufficient published data or original data. All the studies were screened by two reviewers independently to determine whether they satisfied the inclusion and exclusion criteria and discrepancies were resolved by discussion with a third reviewer.

### Data extraction and quality assessment

Two investigators were responsible for data extraction, and the accuracy check was conducted by a third investigator. The BMI categories and RA risk estimates, including relative risk (RR) or odds ratios (ORs) with their 95 % CIs, were regarded as the primary variables of interest. RR or ORs from the maximally adjusted model was used to reduce the possibility of potential residual confounding. For each included study, data including the author name, publication year, study design, the number of patients, age of participants, and gender were extracted. The results were reviewed by two independent reviewers, and any disagreements were resolved by discussion with a third reviewer until a consensus was reached.

### Statistical analysis

According to World Health Organization guidelines (WHO, 2000[36]), individuals with a BMI of 25~30 kg/m^2^ are classified as overweight and those with a BMI ≥ 30 kg/m^2 ^are characterized as obese. This meta-analysis was conducted to compare RA risk in obesity/overweight and normal BMI individuals. Stata Version 12.0 software (Stata Corp, College Station, TX, USA) was used for all statistical analyses. Chi-squared and I^2^ tests were used to determine the significance of heterogeneity and identify the analysis model used for association: fixed-effect or random-effect model (Higgins et al., 2005[16]). A fixed-effect model was used to estimate pooled RRs with 95 % CIs when the Chi-squared test P-value was ≤ 0.05 and I^2^ test-value was > 50 %; otherwise, a random-effect model was selected (DerSimonian and Nan, 1986[7]). Subgroup analyses by sex were performed to investigate the association in male and female subgroups. All categorical data are presented as mean differences and analyzed by OR and 95 % CI. A P value of < 0.05 was considered to be the threshold for statistical significance.

## Results

### Study selection

**Figure 1:**
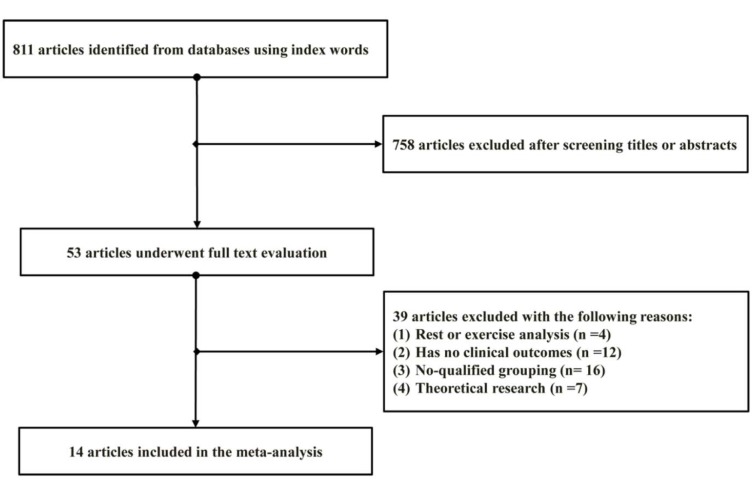
Flow diagram of the literature search and selection process

A total of 811 relevant studies were identified in the initial comprehensive literature search using index words. After screening the title or abstract, 758 articles were discarded as duplicates and 53 articles remained for full-text evaluation. After reviewing the complete manuscript, 39 articles were excluded due to following reasons: rest or exercise analysis (4), without clinical outcomes (12), no-qualified grouping (16), theoretical research (7). Only 14 studies (Crowson et al., 2013[6]; Harpsøe et al., 2014[14]; Lahiri et al., 2014[18]; Ljung and Rantapää-Dahlqvist, 2016[19]; Lu et al., 2014[20]; Palmer and Clegg, 2015[22]; Pedersen et al., 2006[24]; Rodríguez et al., 2009[26]; Sparks et al., 2014[29]; Symmons et al., 1997[30]; Turesson et al., 2016[31]; Uhlig et al., 1999[32]; Voigt et al., 1994[34]; Wesley et al., 2013[35]) that satisfied all of the inclusion criteria were included in this meta-analysis. A flow diagram of this selection process is shown in Figure 1(Fig. 1).

### Characteristics of the included studies

The general characteristics of included studies are summarized in Table 1(Tab. 1) (References in Table 1: Crowson, 2013[6]; Harpsøe, 2014[14]; Lahiri, 2014[18]; Ljung, 2016[19]; Lu, 2014[20]; Palmer, 2015[22]; Pedersen, 2006[24]; Rodríguez, 2009[26]; Sparks 2014[29]; Symmons, 1997[30]; Turesson, 2016[31]; Uhlig, 1999[32]; Voigt, 1994[34]; Wesley, 2013[35]), which includes the first author of the study, publication year, study design, the number of patients, the average age of participants, and gender information. In total, 14 studies with a total of 353,948 patients were included in the analysis. All eligible articles were published in English, and 9 articles were published after 2010. Ten studies were designed as case-control studies and four studies were cohort studies. Five studies reported the average age of both groups, and most patients were aged older than 50 years. Five studies were conducted in female populations and five studies were conducted in male populations, while four studies did not record gender information.

### Overweight and risk of RA

**Figure 2:**
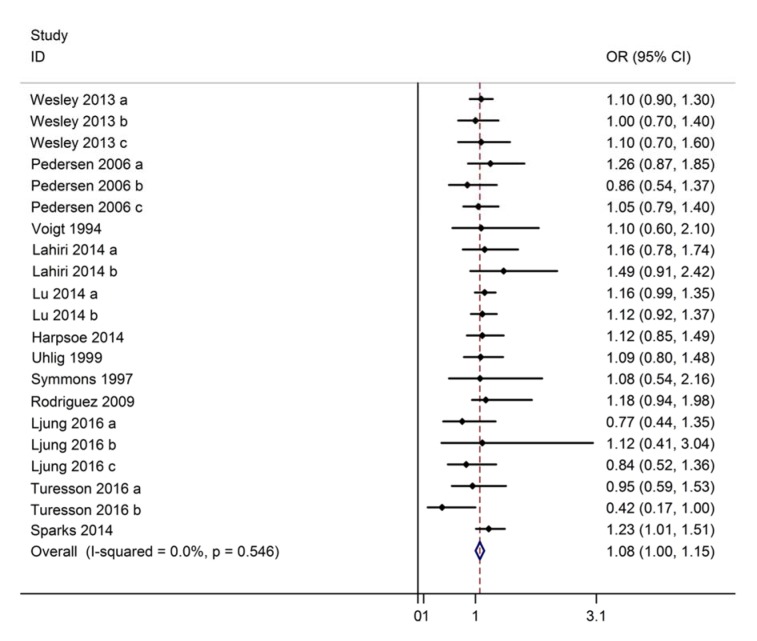
Forest plot showing the risk of rheumatoid arthritis for overweight compared to normal weight

Data from 12 studies (9 case-control studies, 3 cohort studies) with 299,513 participants were pooled to evaluate the association between being overweight and the risk of RA. A fixed-effect model was used to analyze the association based on the Chi-squared test P-value (P = 0.546) and I^2^ test value (I^2^ = 0.0 %). The pooled results suggested that being overweight could lead to a significant increase in RA risk compared with normal weight (OR: 1.08, 95 % CI: 1.00~1.15, Figure 2(Fig. 2); References in Figure 2: Crowson, 2013[6]; Harpsøe, 2014[14]; Lahiri, 2014[18]; Ljung, 2016[19]; Lu, 2014[20]; Palmer, 2015[22]; Pedersen, 2006[24]; Rodríguez, 2009[26]; Sparks 2014[29]; Symmons, 1997[30]; Turesson, 2016[31]; Uhlig, 1999[32]; Voigt, 1994[34]; Wesley, 2013[35]). 

**Figure 3:**
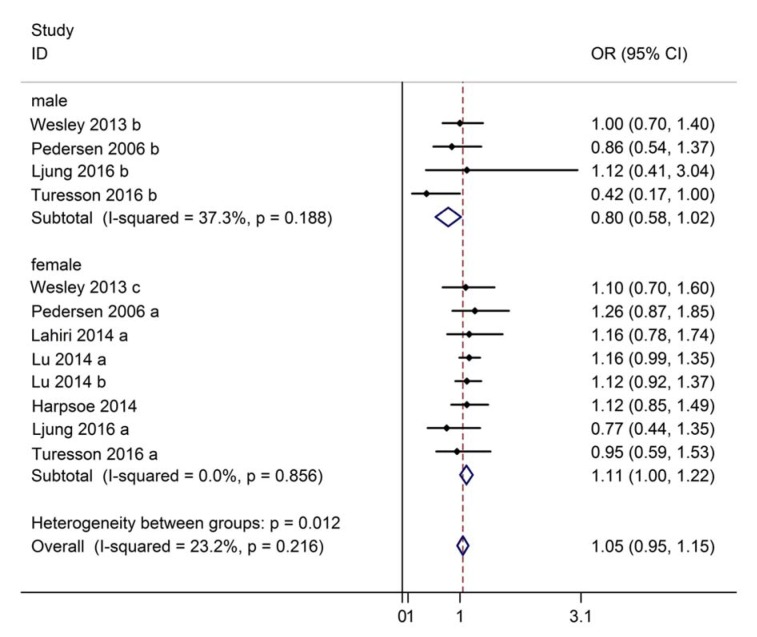
Forest plot showing the risk of rheumatoid arthritis for overweight compared to normal weight in subgroup analyses

In the subgroup analysis, the association of male and female subgroups with RA risk was investigated. Four studies were available for identifying the association between being overweight and risk of RA in males. Significant heterogeneity (I^2^ = 37.3 %, P = 0.188) did not exist, therefore, a fixed-effect model was used. As expected, no significant difference in the risk of RA was found between overweight and normal weight males (OR: 0.80, 95 % CI: 0.58~1.02, Figure 3(Fig. 3); References in Figure 3: Harpsøe, 2014[14]; Lahiri, 2014[18]; Ljung, 2016[19]; Lu, 2014[20]; Pedersen, 2006[24]; Turesson, 2016[31]; Wesley, 2013[35]). Meanwhile, seven studies were available for analyzing the association between being overweight and risk of RA in females. No significant heterogeneity was found (I^2^ = 0.0 %, P = 0.856), and thus a fixed-effect model was used for the analysis. The results indicated that the risk of RA was significantly higher in overweight females than in normal weight females (OR: 1.11, 95 % CI: 1.00~1.22, Figure 3(Fig. 3)), suggesting that being overweight has a positive association with the risk of RA in females.

### Obesity and risk of RA

**Figure 4:**
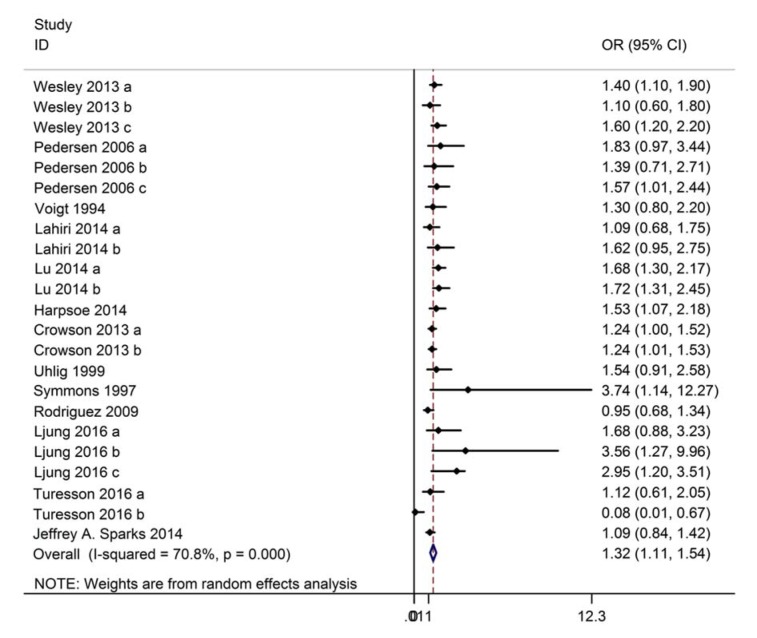
Forest plot showing the risk of rheumatoid arthritis for obesity compared to normal weight

13 studies (10 case-control studies, 3 cohort studies) containing data on 301,139 participants reported the association between obesity and risk of RA. Based on the Chi-squared test P-value (P = 0.000) and I^2 ^tests-value (I^2^ = 70.8 %), a random-effect model was used to analyze the association. Compared to normal weight individuals, obesity significantly increased the risk of RA (OR: 1.32, 95 % CI: 1.11~1.54, Figure 4(Fig. 4); References in Figure 4: Crowson, 2013[6]; Harpsøe, 2014[14]; Lahiri, 2014[18]; Ljung, 2016[19]; Lu, 2014[20]; Palmer, 2015[22]; Pedersen, 2006[24]; Rodríguez, 2009[26]; Sparks 2014[29]; Symmons, 1997[30]; Turesson, 2016[31]; Uhlig, 1999[32]; Voigt, 1994[34]; Wesley, 2013[35]). 

**Figure 5:**
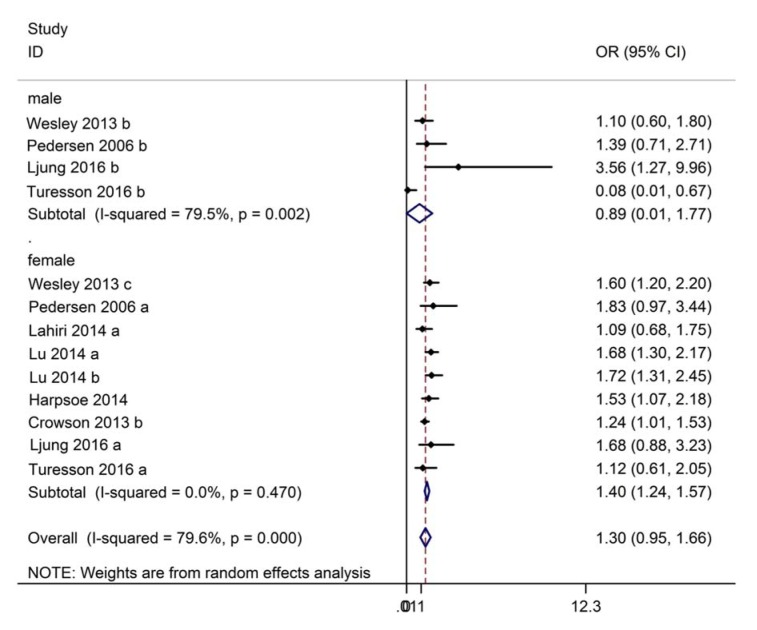
Forest plot showing the risk of rheumatoid arthritis for obesity compared to normal weight in subgroup analyses

In the subgroup analysis, the association of male and female subgroups with RA risk was evaluated. Four studies reported the association between obesity and risk of RA in males. There was significant heterogeneity (I^2^ = 79.5 %, P = 0.002), therefore the association was assessed using a random-effect model. No significant difference in the risk of RA (OR: 0.89, 95 % CI: 0.01~1.77, Figure 5(Fig. 5); References in Figure 5: Crowson, 2014[6]; Harpsøe, 2014[14]; Lahiri, 2014[18]; Ljung, 2016[19]; Lu, 2014[20]; Pedersen, 2006[24]; Turesson, 2016[31]; Wesley, 2013[35]) was found between obese and normal weight males. Eight studies were available for investigating the association between obesity and risk of RA in females. No significant heterogeneity was seen (I^2^ = 0.0 %, P = 0.470), therefore, a fixed-effect model was applied. In the pooled results, a significantly higher risk of RA was found in obese females compared to normal weight females (OR: 1.40, 95 % CI: 1.24~1.57, Figure 5(Fig. 5)), suggesting that obesity may increase the risk of RA, especially in females.

## Discussion

Rheumatoid arthritis (RA) is a common chronic autoimmune disease and has been shown to be the consequence of a combination of genetic and environmental factors. Environmental risks including age, gender, obesity, cigarette smoking, alcohol consumption, and dietary habits, have been shown to be closely associated with the occurrence of this disorder (Di et al., 2014[8]; Jin et al., 2014[17]; Parks et al., 2013[23]; Voigt et al., 1994[34]). As an important potential risk factor, obesity and its association with RA development has been explored in several previous studies; however, the exact relationship remains poorly understood. In this study, we conducted a meta-analysis to confirm the association of BMI with RA development based on the current literature. The findings of this study indicated that BMIs indicating overweight or obesity are risk factors for RA. Furthermore, women with high BMI are more likely to suffer from RA compared than men.

Twelve studies reporting the association between BMI with RA risk revealed a positive association between overweight BMIs and RA when pooled (OR: 1.08, 95 % CI: 1.00~1.15). Further subgroup analysis showed that the OR for RA was higher in females (OR: 1.11, 95 % CI: 1.00~1.22) compared to males (OR: 0.80, 95 % CI: 0.58~1.02). Meanwhile, thirteen studies reported the association between obesity and risk of RA, and similar results were obtained. Collectively, it can be concluded that being overweight or obese can lead to a higher risk of RA development. In addition, the incidence of RA in women with high BMI is much higher than that in men. Compared with the findings of recent research, the results of this meta-analysis are similar. Qin et al. (2015[25]) found a significantly increased risk of RA in the categories of obese and overweight populations compared with individuals with normal weight. Further dose-response analysis indicated that there was a nonlinear association between the high levels of BMI and RA development, and that the estimated summary relative risk for a 5-unit increment was 1.03 (95 % CI: 1.01 ~1.05). Feng et al. (2016[11]) reported that the RR of RA in obesity cases was 16 % more than that in overweight cases, meanwhile, the risk of RA increased by 13 % for every 5 kg/m^2^ increase in BMI. Further subgroup analyses showed that the effect of BMI on the occurrence of RA was related to sex and serological status. Notably, overweight/ obese females and seronegative individuals were more likely to develop RA when compared to those with normal BMI. Even though the exact mechanism by which an increased risk of RA occurs in overweight/obese individuals remains unclear, there are several plausible explanations. First, obesity may strengthen the autoimmune response through multiple mechanisms, such as increasing the secretion of adipokines (Versini et al., 2014[33]). Second, obesity could modify the influence of sex hormones on the development of RA (Doran et al., 2004[9]). Third, gene variation can also change the relationship between obesity and autoimmune diseases (Soltani-Arabshahi et al., 2010[28]). However, follow-up studies are needed for verification of these hypotheses.

The major strengths of this meta-analysis were as follows: First, this meta-analysis was based on a comprehensive literature search of several databases to identify all relevant comparative studies. Therefore, a very large number of RA cases were included in this study, which improved the reliability, statistical power, and validity of our results. Second, most of the included studies were published in relatively high impact journals in recent years, which were of high-quality and contained more comprehensive content. Despite these strengths, several potential limitations also should also be noted. First, the included studies were limited to the English language and some qualified studies published in other languages were excluded, as were some unpublished data; thus, some publication bias existed. Second, the study populations of the included studies were limited to North American and European populations, therefore data for other continents are lacking. The development of RA is influenced by lifestyle, living environment, and economic development, thus, obesity or increased BMI may have different effects on the incidence rate of this disease in different places around the world. Based on the above, further investigations should be performed to evaluate the association between BMI and RA in different regions of the world, such as Africa and Asia. Third, RA is a multifactorial disease governed by complex gene-environment interactions and few researchers have reported on the influence of genetic factors. Therefore, we were unable to assess the effects of genetic factors on this disease. Fourth, further sensitivity analyses and publication bias analyses were unable to be performed in this study due to the lack of detailed information from the individual studies.

This meta-analysis systematically assessed the association between body mass index (BMI) and rheumatoid arthritis (RA) risk based on the relevant current literature. The results demonstrated that increased BMI (classified as overweight or obese) is associated with a higher risk of developing RA. Furthermore, the positive association between BMI and RA risk may be stronger in females than in males, emphasizing sex dependency of RA. Despite these results, future work is needed to combine more risk factors in large prospective studies to reveal the mechanisms of gene-environment interactions in the development of RA. 

## Acknowledgements

None.

## Conflict of interest

We all declare that we have no conflict of interest.

## Figures and Tables

**Table 1:**
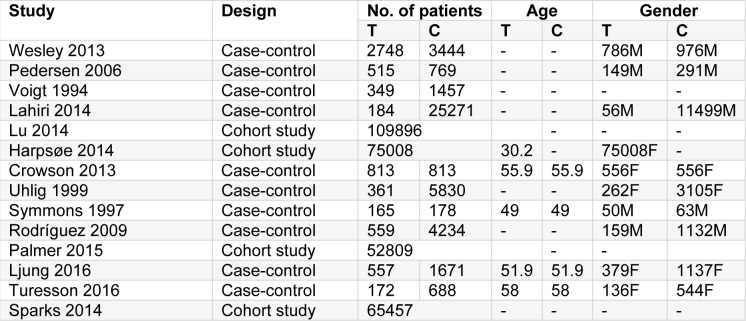
Basic characteristics of the included studies
